# Elevated Circulating Levels of Inflammatory Markers in Patients with Acute Coronary Syndrome

**DOI:** 10.1155/2015/805375

**Published:** 2015-10-04

**Authors:** Hamad Al Shahi, Kazunori Shimada, Katsumi Miyauchi, Takuma Yoshihara, Eiryu Sai, Tomoyuki Shiozawa, Ryo Naito, Tatsuro Aikawa, Shohei Ouchi, Tomoyasu Kadoguchi, Tetsuro Miyazaki, Hiroyuki Daida

**Affiliations:** Department of Cardiovascular Medicine, Juntendo University Graduate School of Medicine, 2-1-1 Hongo, Bunkyo-ku, Tokyo 113-8421, Japan

## Abstract

*Objective*. We evaluated inflammatory cytokines and chemokine in peripheral blood mononuclear cells (PBMCs) in patients with either acute coronary syndrome (ACS) or stable coronary artery disease (CAD). *Methods*. We enrolled 20 ACS patients and 50 stable CAD patients without previous history of ACS who underwent cardiac catheterization. Patients with an estimated glomerular filtration rate of ≤30 mL/min/1.73 m^2^ and C-reactive protein of ≥1.0 mg/dL were excluded. Blood samples were collected from the patients just before catheterization, and PBMCs were isolated from the whole blood. The levels of inflammatory cytokines and chemokine were measured by using real-time quantitative polymerase chain reaction and immunoassays. *Results*. The expression of tumor necrosis factor alpha (TNF-*α*), interleukin- (IL-) 6, IL-10, IL-23A, IL-27, and IL-37 was significantly higher in the ACS group than in the CAD group (*P* < 0.05). In contrast, the expression of IL-33 was significantly lower in the ACS group than in the CAD group (*P* < 0.05). The ACS patients had higher plasma levels of TNF-*α*, IL-6, and IL-10 in the ACS group than in the CAD group. *Conclusion*. Circulating levels of pro-/anti-inflammatory cytokines, including IL-23A, IL-27, IL-33, and IL-37, may be associated with the pathogenesis of atherosclerosis in ACS patients.

## 1. Introduction

Cardiovascular diseases (CVDs) are the leading cause of morbidity and mortality worldwide [1]. Atherosclerosis is a major cause of CVDs, including myocardial infarction, stroke, heart failure, and peripheral artery disease [2]. Acute coronary syndrome (ACS) encompasses a spectrum of unstable coronary artery diseases (CADs), including unstable angina pectoris (UAP) and acute myocardial infarction (AMI), which occur in response to vascular inflammation, plaque rupture, and subsequent thrombosis [3].

Inflammatory markers, such as C-reactive protein (CRP), inflammatory cytokines, and chemokines, have been implicated in the initiation and progression of CAD [4]. Interleukin- (IL-) 12 and IL-23 are members of a small family of proinflammatory heterodimeric cytokines that share a common p40 subunit that is covalently linked either to a p35 subunit to form IL-12 or to a p19 subunit to form IL-23A [5]. Another member of the IL-12 family, IL-27, has also been identified [6]. The increased expression of IL-27 has been associated with the sites of inflammation in patients with autoimmune diseases, such as those with Crohn's disease [7]. IL-33 was originally described as a modulator of inflammation that switched the balance of T-helper 1 (Th1) to Th2 mediated immune responses [8]. In apoE knockout mice on a high-fat diet, IL-33 has been shown to reduce atherosclerosis development [8, 9]. IL-37, also known as IL-1F7, is a newly discovered anti-inflammatory cytokine expressed in several tissues and in inflammatory cells [8].* In vitro*, the induction of IL-37 in epithelial cells or macrophages has been shown to almost completely suppress the production of proinflammatory cytokines, including tumor necrosis factor alpha (TNF-*α*), IL-1*α*, and IL-1*β* [8, 10].

In the present study, we aimed to evaluate the expression profiles of inflammatory cytokines and chemokine, including TNF-*α*, IL-6, IL-10, IL-18, IL-18 binding protein (IL-18BP), IL-23A, IL-27, IL-33, IL-37, and monocyte chemoattractant protein- (MCP-) 1 in peripheral blood mononuclear cells (PBMCs) of patients with either ACS or stable CAD.

## 2. Methods

### 2.1. Study Population

The study protocol conformed to the principles of the Declaration of Helsinki and was performed with approval of the Ethics Committee of Juntendo University. All subjects gave informed consent, both verbal and written, for participation in the study, and underwent coronary artery angiography at Juntendo University Hospital. A total of 70 consecutive patients (mean age: 69 years) were enrolled. The participants were divided into two groups; the stable CAD group (*n* = 50) and ACS group (*n* = 20). ACS was defined as a spectrum of clinical conditions and was subdivided into the UAP group (*n* = 5) and the AMI group (*n* = 15), with the latter including ST-segment elevation myocardial infarction (STEMI) and non-STEMI. The exclusion criteria were previous history of ACS and autoimmune, neoplastic, liver, and hematological diseases. Patients with an estimated glomerular filtration rate (eGFR) of ≤30 mL/min/1.73 m^2^ and CRP of ≥1.0 mg/dL were also excluded.

### 2.2. Blood Samples

Blood samples were collected from the patients just before catheterization. The samples were collected into sodium heparin tubes and the fresh PBMCs were purified using standard Ficoll centrifugation according to the instructions of the manufacturer (Axis-Shield POC, Oslo, Norway). Briefly, 4 mL of Ficoll was pipetted into two 15 mL centrifuge tubes. The heparinized blood was diluted in the ratio 1 : 1 in phosphate-buffered saline (PBS) and was carefully layered over the Ficoll (9-10 mL/tube). The tubes were centrifuged for 20 min at 1,020 ×g. The cell interface layer was harvested carefully, and the cells were washed twice in PBS (for 10 min at 640 ×g followed by 10 min at 470 ×g) and resuspended in RPMI 1640 medium before counting [11, 12]. The plasma samples were stored at −80°C until further use.

### 2.3. Real-Time Quantitative Polymerase Chain Reaction (RT-qPCR) to Determine the mRNA Expression Levels of Biomarkers

To investigate the mRNA expression levels of inflammatory cytokines and chemokine, including TNF-*α*, IL-6, IL-10, IL-18, IL-18-BP, IL-23A, IL-27, IL-33, IL-37, and MCP-1, total RNA was isolated from the fresh PBMCs using RNeasy Mini kit + QiaShredder spin columns (Qiagen, Valencia, CA, USA). In brief, the PBMCs were disrupted in the RLT buffer and homogenized. Ethanol was added and the mixture was applied to an RNeasy Mini spin column. Then, the total RNA was washed using buffer RW1 and buffer RPE, containing ethanol. Finally, the total RNA was eluted in RNase-free water. Quality was determined using NanoDrop 2000 spectrophotometer (Wilmington, USA). cDNA was synthesized using a High Capacity cDNA Reverse Transcription Kit (Applied Biosystems, Foster City, CA, USA). RT-qPCR was performed using SYBR Premix Ex Taq II (Takara Biotechnology, Japan) on a 7500 Real-Time PCR system (Applied Biosystems, Foster City, CA) following the manufacturer's instructions. The specific primers used are presented in Table 1, and gene expression levels (amount of target, normalized to endogenous control gene) were calculated using standard curve method.

### 2.4. Measurement of Marker Concentration Levels by High-Sensitivity Assay

The plasma concentration levels of TNF-*α*, MCP-1, IL-6, and IL-10 were determined using commercial multiplexed fluorescent bead-based immunoassays (Milliplex Map Kit, EMD Millipore Co., Billerica, MA) and were measured by a Luminex 200 xPONENT 3.1 System (EMD Millipore Co., Billerica, MA). In the experimental design, the base kit can be used with any combination of the four analyte-specific bead sets for greater flexibility. The minimal detectable concentration levels were 1.4 pg/mL for TNF-*α*, 144.5 pg/mL for MCP-1, 0.2 pg/mL for IL-6, and 0.5 pg/mL for IL-10. All of the samples were measured in duplicate. The assays were performed according to the manufacturer's instructions.

### 2.5. Statistical Analysis

Statistical analysis was performed by using Prism software (GraphPad Software, San Diego, USA). Data were compared using Student's *t*-test or Mann-Whitney test, depending on the data distribution. For more than two groups, one-way analysis of variance was performed for multiple comparisons followed by* post hoc* Tukey's procedure for multiple range tests. Results were considered significant when the *P* value was <0.05.

## 3. Results

### 3.1. Clinical Characteristics of the Patients

The clinical characteristics and laboratory measurements of the patients are summarized in Table 2. There were no significant differences between the two groups in the following variables: age, sex, the presence of risk factors, and medications. The levels of total cholesterol, triglycerides, and low-density lipoprotein (LDL) cholesterol were significantly higher in the ACS group than in the CAD group (*P* < 0.05). The other parameters showed no significant differences between the two groups.

### 3.2. Expressions of Inflammatory Markers in PBMCs

**Figure 1:**
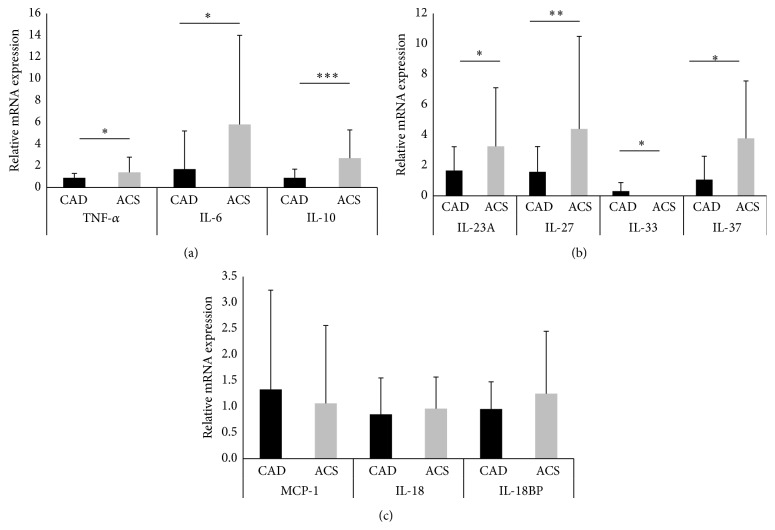
The data are presented as means ± SD. Profile of the expression levels of tumor necrosis factor-*α* (TNF-*α*), interleukin- (IL-) 6, and IL-10 (a); IL-23A, IL-27, IL-33, and IL-37 (b); and monocyte chemoattractant protein-1 (MCP-1), IL-18, and IL-18 binding protein (BP) (c) in the coronary artery disease (CAD) and acute coronary syndrome (ACS) groups. ^*∗*^ *P* < 0.05; ^*∗∗*^ *P* < 0.01; ^*∗∗∗*^ *P* < 0.001.

**Figure 2:**
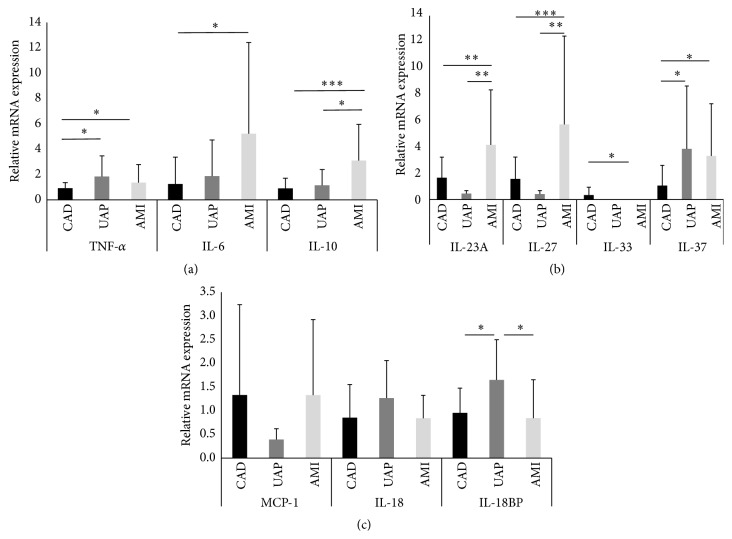
The data are presented as means ± SD. Profile of the expression levels of tumor necrosis factor-*α* (TNF-*α*), interleukin- (IL-) 6, and IL-10 (a); IL-23A, IL-27, IL-33, and IL-37 (b); and monocyte chemoattractant protein-1 (MCP-1), IL-18, and IL-18 binding protein (BP) (c) in the coronary artery disease (CAD), unstable angina pectoris (UAP), and acute myocardial infarction (AMI) groups. ^*∗*^ *P* < 0.05; ^*∗∗*^ *P* < 0.01; ^*∗∗∗*^ *P* < 0.001.

The expression levels of inflammatory markers are summarized in Figure 1. The expression levels of TNF-*α* (1.4 ± 1.4 versus 0.9 ± 0.4, *P* < 0.05), IL-6 (4.2 ± 6.2 versus 1.2 ± 2.1, *P* < 0.05), IL-10 (2.5 ± 2.6 versus 0.9 ± 0.8, *P* < 0.001), IL-23A (3.1 ± 3.9 versus 1.6 ± 1.5, *P* < 0.05), IL-27 (4.2 ± 6.1 versus 1.5 ± 1.6, *P* < 0.01), and IL-37 (3.4 ± 3.8 versus 1.0 ± 1.5, *P* < 0.05) were significantly higher in the ACS group than in the CAD group. However, the expression levels of IL-33 were significantly lower in the ACS group (0.3 ± 0.5 versus 0.003 ± 0.003, *P* < 0.05) than in the CAD group. The expression levels of MCP-1 (1.0 ± 1.4 versus 1.3 ± 1.9, *P* = 0.59), IL-18 (0.9 ± 0.5 versus 0.8 ± 0.7, *P* = 0.60), and IL-18BP (1.4 ± 0.8 versus 0.9 ± 0.5, *P* = 0.59) showed no significant differences between the two groups. Further analysis of the CAD, UAP, and AMI groups was performed and the results are summarized in Figure 2. In the three groups, the expression levels of TNF-*α* were significantly higher in the AMI and UAP groups than in the CAD group (AMI, 1.3 ± 1.4; UAP, 1.8 ± 1.6; CAD, 0.9 ± 0.4). The expression levels of IL-6 were significantly higher in the AMI group than in the CAD group (AMI, 5.2 ± 7.2; UAP, 1.8 ± 2.8; CAD, 1.2 ± 2.1). The expression levels of IL-10 were significantly higher in the AMI group than in the UAP and CAD groups (AMI, 3.1 ± 2.8; UAP, 1.1 ± 1.2; CAD, 0.9 ± 0.8). The expression levels of IL-23A and IL-27 were significantly higher in the AMI group than in the UAP and CAD groups (IL-23A; AMI, 4.1 ± 4.1; UAP, 0.4 ± 0.2; CAD, 1.6 ± 1.5; IL-27; AMI, 5.7 ± 6.7; UAP, 0.4 ± 0.2; CAD, 1.5 ± 1.6). The expression levels of IL-37 were significantly higher in the AMI and UAP groups than in the CAD group (AMI, 3.3 ± 3.9; UAP, 3.8 ± 4.7; CAD, 1.0 ± 1.5). However, the expression of IL-33 was significantly lower in the AMI group than in the CAD group (AMI, 0.002 ± 0.002; UAP, 0.01 ± 0.01; CAD, 0.34 ± 0.60). The expression levels of IL-18BP were significantly higher in the UAP group than in the AMI and CAD groups (AMI, 0.8 ± 0.8; UAP, 1.6 ± 0.8; CAD, 0.9 ± 0.5). There were no significant differences in the expression levels of MCP-1 and IL-18 between the three groups (MCP-1; AMI, 1.3 ± 1.5; UAP, 0.3 ± 0.2; CAD, 1.3 ± 1.9, IL-18; AMI, 0.8 ± 0.4; UAP, 1.2 ± 0.7; CAD, 0.8 ± 0.7).

### 3.3. Measurement of Marker Concentration Levels by High-Sensitivity Assay

**Figure 3:**
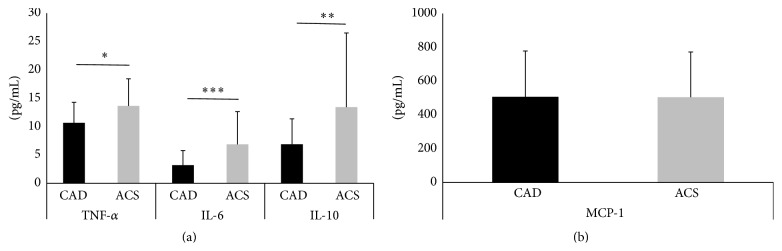
The data are presented as means ± SD. Profile of plasma levels of tumor necrosis factor-*α* (TNF-*α*), interleukin- (IL-) 6, and IL-10 (a) and of monocyte chemoattractant protein-1 (MCP-1) (b) in coronary artery disease (CAD) and acute coronary syndrome (ACS) groups. ^*∗*^ *P* < 0.05; ^*∗∗*^ *P* < 0.01; ^*∗∗∗*^ *P* < 0.001.

**Figure 4:**
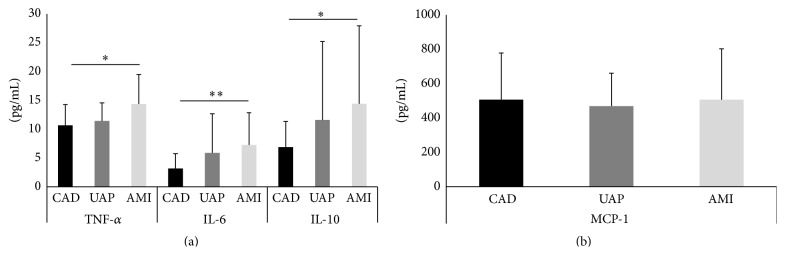
The data are presented as means ± SD. Profile of plasma levels of tumor necrosis factor-*α* (TNF-*α*), interleukin- (IL-) 6, and IL-10 (a) and of monocyte chemoattractant protein-1 (MCP-1) (b) in the coronary artery disease (CAD), unstable angina pectoris (UAP), and acute myocardial infarction (AMI) groups. ^*∗*^ *P* < 0.05; ^*∗∗*^ *P* < 0.01.

The plasma levels of inflammatory markers are summarized in Figure 3. The plasma concentration levels (pg/mL) of TNF-*α*, IL-6, and IL-10 were higher in the ACS group than in the CAD group (TNF-*α*; 13.6 ± 4.7 versus 10.6 ± 3.6, *P* < 0.05; IL-6; 6.8 ± 5.7 versus 3.1 ± 2.5, *P* < 0.001; IL-10; 13.4 ± 13.0 versus 6.8 ± 4.4, *P* < 0.01). However, there were no significant differences in the plasma levels (pg/mL) of MCP-1 between the ACS and CAD groups (496.1 ± 266.4 versus 506.8 ± 271.1, *P* = 0.88). Further analysis of the CAD, UAP, and AMI groups was conducted and the results are summarized in Figure 4. Within the three groups, the plasma levels (pg/mL) of TNF-*α*, IL-6, and IL-10 were significantly higher in the AMI group than in the CAD group (TNF-*α*; AMI, 14.1 ± 5.0; UAP, 11.4 ± 3.1; CAD, 10.6 ± 3.6; IL-6; AMI, 6.9 ± 5.5; UAP, 5.8 ± 6.7; CAD, 3.1 ± 2.5; IL-10; AMI, 13.4 ± 13.1; UAP, 11.6 ± 13.5; CAD, 6.8 ± 4.4). There were no significant differences in the plasma levels (pg/mL) of MCP-1 between the three groups (AMI, 487.4 ± 293.6; UAP, 469.1 ± 191.3; CAD, 506.8 ± 271.1).

## 4. Discussion

This study showed that the expression and plasma levels of the pro- and anti-inflammatory mediators were dramatically higher in patients with ACS than in patients with CAD. The results suggested that IL-23A, IL-27, IL-33, and IL-37 could be used as novel index values of the two diseases and as diagnostic indicators of ACS.

The evidence from the present study indicated that the expression levels of TNF-*α* and IL-6 were significantly higher in the ACS group than in the CAD group. TNF-*α* and IL-1 stimulate smooth muscle cells to produce IL-6, which is mainly expressed in human atherosclerotic lesions [13, 14]. The high levels of TNF-*α* and IL-6 observed in the ACS patients in this study were comparable to those in other studies [15, 16].

The gene encoding the IL-23 p19 protein, later named IL23A, was first identified in 2000 by Oppmann et al. who were able to build a disulfide-bridged complex with the p40 subunit originally described for IL-12 [5]. IL-23A functions as a proinflammatory subunit of the heterodimeric cytokine, IL-23, which is expressed and produced by macrophages and dendritic cells (DCs) [17]. IL-23A is involved in the inflammatory response through enhancing matrix metalloprotease 9 by stimulating angiogenesis and reducing CD8^+^ T-cells infiltration [17, 18]. In this study, IL-23A expression levels were significantly higher in the patients with ACS than in the CAD patients. Considering the ACS subgroups, the IL-23A expression levels were significantly higher in the patients with AMI than in those with either UAP or CAD, which indicated that this inflammatory response is related to myocardial damage as shown in particular in the AMI patients.

IL-27 is another heterodimeric cytokine member of the IL-12 family and is composed of the p28 subunit and p40-related protein Epstein-Barr-virus-induced gene 3 [19]. IL-27 was originally described as a proinflammatory cytokine produced by human phagocytic cells and DCs. IL-27 induces proliferation of naive T cells and regulates the commitment of CD4^+^ T lymphocytes to Th1 differentiation [19, 20]. Although the activity of IL-27 is well characterized in animal immune cells, little information is available regarding its effects on human immune cells. In this study, the expression levels of IL-27 were significantly higher in patients with ACS than in the CAD patients. Considering the ACS subgroups, IL-27 expression levels were significantly higher in the patients with AMI than in those with either UAP or CAD. According to previous reports, IL-27 promoted early differentiation of Th1 and demonstrated significant elevation in the percentage of circulating Th1 cells in myocardial damage [21, 22].

IL-33 (also called IL-1F11) is member of the IL-1 family, which induces Th2-type responses, and has been recognized to inhibit the inflammatory response in atherosclerosis by stimulating a Th1-to-Th2 switch and increasing the number of regulatory T cells [9, 23]. The immunological response of Th1 is believed to accelerate atherosclerosis, whereas the response of Th2 type reduces the progression of atherosclerosis [23]. This study showed that expression of IL-33 was significantly higher in the patients with CAD than in those with ACS, especially in the AMI patients, which suggests that in CAD patients IL-33 has a protective role against progression to ACS.

IL-37 belongs to the IL-1 cytokine family and functions as a natural suppressor of innate inflammatory and immune responses [10]. Increased IL-37 levels have been associated with many autoimmune and chronic inflammatory diseases, such as rheumatoid arthritis, systemic lupus erythematosus, and Guillain-Barré syndrome in humans [24–26]. The present study showed that the expression levels of IL-37 were higher in ACS (UAP and AMI) than in CAD patients, which indicated that an increase in IL-37 levels may be caused by excessive inflammatory response in ACS.

In ACS patients, low expression levels of IL-10 in serum samples have been associated with an increased risk of cardiovascular events and high IL-10 expression levels have been associated with a decreased risk [27, 28]. However, conflicting results have been published [29]. We have previously reported that patients with unstable angina had significantly lower blood IL-10 concentrations than those with stable angina [11]. This finding suggests that lower IL-10 levels are associated with greater clinical instability and supports the hypothesis that IL-10 has a protective role in atherogenesis by maintaining plaque stability. However, AMI with myocardial necrosis is associated with a greater inflammatory response than unstable angina is. Unstable angina is associated with an inflammatory mechanism unrelated to the presence of necrosis and with extension of coronary lesions [30]. The present study showed that patients with ACS, especially AMI patients, had higher IL-10 expression levels than CAD patients had. Elevation of IL-10 in the AMI patients was correlated with systemic proinflammatory activity, as evaluated by plasma concentrations of TNF-*α* and IL-6, which may be related to thrombosis, plaque rupture, and cardiac damage.

In this study, there were no significant differences in the MCP-1 expression levels and plasma concentration levels between the patient groups. In population studies, plasma levels of MCP-1 have been shown to be positively correlated with cardiovascular risk factors, measures of coronary atherosclerosis burden, and incident coronary and peripheral artery disease [31, 32]. There were also no significant differences in the expression levels of IL-18 and IL-18BP between the ACS and CAD groups. However, among the subgroups, higher IL-18BP expression levels were shown in the UAP patients than in the AMI and CAD patients. IL-18 is a member of the IL-1 family of cytokines and is an important cytokine that promotes Th1 and natural killer cell activity, so IL-18 is likely to have a key role in atherosclerotic plaque instability [33]. The activity of IL-18 is balanced by the presence of the high-affinity, naturally occurring cytokine, IL-18BP. IL-18BP avidly binds IL-18 and has been shown to reduce production of inflammatory cytokines induced by IL-18* in vitro* and* in vivo*, which suggests that the role of IL-18BP in inflammatory regulation is complex [34, 35]. Although some studies have found that IL-18 levels were significantly elevated in ACS patients, further studies are needed to investigate the relationship between IL-18 levels and prognosis of CAD.

## 5. Limitation

There were some limitations to this study. First, the number of subjects was small. Second, we were unable to obtain Milliplex assay data for all inflammatory markers. Third, this study relied on a single baseline blood sample; thus, we could not assess potential variation in the inflammatory marker levels over time.

## 6. Conclusion

This study showed that circulating levels of proinflammatory cytokines and chemokine might be involved in the triggering stage of ACS. Further studies are needed to confirm their precise role in the pathogenesis of this syndrome.

## Figures and Tables

**Table 1:** Primer sequences for real-time quantitative polymerase chain reaction.

Gene	Primer pair sequences (5′-3′)
ACTB (F)	5′-TGGCACCCAGCACAATGAA-3′
ACTB (R)	5′-CTAAGTCATAGTCCGCCTAGAAGCA-3′

TNF-*α* (F)	5′-TGCTTGTTCCTCAGCCTCTT-3′
TNF-*α* (R)	5′-CAGAGGGCTGATTAGAGAGAGGT-3′

MCP-1 (F)	5′-AGCAGCAAGTGTCCCAAAGA-3′
MCP-1 (R)	5′-GGTGGTCCATGGAATCCTGA-3′

IL-6 (F)	5′-GCCAGAGCTGTGCAGATGAG-3′
IL-6 (R)	5′-TCAGCAGGCTGGCATTTG-3′

IL-10 (F)	5′-GAGATGCCTTCAGCAGAGTGAAGA-3′
IL-10 (R)	5′-AAGGCTTGGCAACCCAGGTA-3′

IL-18 (F)	5′-GACCTTCCAGATCGCTTCCTC-3′
IL-18 (R)	5′-GATGCAATTGTCTTCTACTGGTTCA-3′

IL-18BP (F)	5′-CATGACCATGAGACACAACTGGA-3′
IL-18BP (R)	5′-AGGTGTGGCTCTGACCAGGA-3′

IL-23A (F)	5′-ATCCAGTGTGGAGATGGCTGTG-3′
IL-23A (R)	5′-AAATCACACCCTGGTGGATCCTT-3′

IL-27 (F)	5′-GGAGTTCACAGTCAGCCTGCAT-3′
IL-27 (R)	5′-CACTCCTGGCAGGTGAGATTC-3′

IL-33 (F)	5′-TGCATGCCAACAACAAGGA-3′
IL-33 (R)	5′-TCCAGGATCAGTCTTGCATTC-3′

IL-37 (F)	5′-TCATCCTTGAGCTCAGCCTCT-3′
IL-37 (R)	5′-GCAGCCAGCTTCATCAGTTTC-3′

**Table 2:** Clinical characteristics of patients.

Characteristics	CAD	ACS
	(*N* = 50)	(*N* = 20)
Age (years)	70 ± 7	65 ± 11
Sex (male/female)	40/10	19/1
Risk factors		
Family history, *n* (%)	13 (26)	6 (30)
Hypertension, *n* (%)	38 (76)	12 (60)
Diabetes, *n* (%)	21 (42)	5 (25)
Smoking, *n* (%)	35 (70)	13 (65)
Body mass index	24.5 ± 3.7	24.1 ± 2.5
Systolic BP (mm Hg)	138 ± 19	139 ± 25
Diastolic BP (mm Hg)	78 ± 12	81 ± 17
Laboratory measurements		
TC (mg/dL)	165 ± 28	189 ± 44^*∗*^
TG (mg/dL)	120 ± 48	155 ± 106^*∗*^
LDL cholesterol (mg/dL)	96 ± 26	114 ± 34^*∗*^
HDL cholesterol (mg/dL)	45 ± 13	48 ± 10
Creatinine (mg/dL)	0.84 ± 0.22	0.77 ± 0.26
HbA1c (%)	5.8 ± 0.7	5.7 ± 0.8
Hs-CRP (mg/dL)	0.12 ± 0.16	0.13 ± 0.07
BNP (pg/mL)	57 ± 72	89 ± 121
Medications, *n* (%)		
Antiplatelet	50 (100)	20 (100)
*β*-blocker	24 (48)	6 (30)
ACEI/ARB	27 (54)	11 (55)
CCB	23 (46)	6 (30)
Statin	32 (64)	12 (60)
Antianginal drug	16 (32)	2 (10)

The data are presented as mean ± SD. CAD: coronary artery disease; ACS: acute coronary syndrome; BP: blood pressure; TC: total cholesterol; TG: triglycerides; LDL: low-density lipoprotein; HDL: high-density lipoprotein; Hb: hemoglobin; Hs-CRP: high-sensitivity C-reactive protein; BNP: brain natriuretic peptide; ACEI: angiotensin-converting enzyme inhibitor; ARB: angiotensin receptor blocker; CCB: calcium channel blocker. ^*∗*^ *P* < 0.05.
